# A New Animal Model of Laryngeal Transplantation

**DOI:** 10.3390/jcm11216427

**Published:** 2022-10-30

**Authors:** Pierre Philouze, Olivier Malard, Sébastien Albert, Lionel Badet, Bertrand Baujat, Frédéric Faure, Carine Fuchsmann, Franck Jegoux, Jean Lacau-St-Guily, Jean-Paul Marie, Antoine Ramade, Sebastien Vergez, Philippe Ceruse, Olivier J. Gauthier

**Affiliations:** 1 Département d’ORL et Chirurgie Cervico-Faciale, Croix-Rousse Hospital, Université Lyon 1, 69004 Lyon, France; 2 Service d’ORL, Hôtel-Dieu, Université Nantes, 44093 Nantes, France; 3 Service d’ORL, Hôpital Bichat, University Paris VII, AP-HP, 75018 Paris, France; 4 Service de Chirurgie de la Transplantation et d’Urologie, Hôpital Edouard-Herriot, Université Lyon 1, 69347 Lyon, France; 5 Service d’ORL et Chirurgie Cervico-Faciale, Hôpital Tenon, APHP, Sorbonne Université, 75013 Paris, France; 6 Département d’ORL et Chirurgie Cervico-Faciale, Hôpital Edouard Herriot, Université Lyon 1, 69100 Lyon, France; 7 Service d’ORL et Chirurgie Maxillo-Faciale, CHU Pontchaillou, Université Rennes, 35033 Rennes, France; 8 Service ORL et Chirurgie Cervico-Faciale, Hôpital Charles-Nicolle, CHU Rouen, Université Rouen Normandie, UR 3830 GRHVN, 76000 Rouen, France; 9 Service ORL et Chirurgie Cervicofaciale, CHU Toulouse Rangueil-Larrey, Université Toulouse, 31059 Toulouse, France; 10 Department of Small Animal Surgery and Anesthesia, Nantes-Atlantic College of Veterinary Medicine, Food Science and Engineering (ONIRIS), 44307 Nantes, France

**Keywords:** larynx, allotransplantation, model, preclinical, laryngeal transplantation, allograft

## Abstract

Only three laryngeal transplants have been described in the literature to date, and none of the techniques has enabled a completely satisfactory functional result to be obtained. This article presents a new model of laryngeal transplantation, with quality of revascularisation of the transplant being the principal objective and optimisation of the various steps of the procedure, with the integration of a new reinnervation technique as a secondary objective. We present a preclinical animal study. Three pig larynges removed in vivo underwent allotransplantation according to the same protocol. The quality of the revascularisation was examined immediately after the surgery as well as by endoscopy for one animal on the fourth day after the operation. The mean time of cold ischaemia was 3 h 15 min. The anaesthetic tolerance of the pigs was excellent. Revascularisation was achieved and judged to be excellent for the three transplants immediately after the operation and the endoscopy performed for one pig on the fourth day after the operation confirmed this result. The anatomical similarities also enabled the application and integration of an innovative technique of laryngeal reinnervation into the various phases of the operation. We describe a reliable and reproducible animal model for laryngeal transplantation. Its application in humans can be envisaged.

## 1. Introduction

Since the 1960s, much progress has been made in the field of organ transplantation, which enables transplants to be performed today in the treatment of handicaps; hand and face transplants, for example, enter into this field of application [1,2]. Despite this progress, there are scarce data concerning laryngeal transplantation and, to our knowledge, only few cases have been published in the English language literature [3,4].

The first larynx allotransplant dates to 1969 and was performed by Kluyskens on a patient who had had a total laryngectomy due to epidermoid carcinoma [5]. The patient no longer required tracheotomy and survived eight months until relapse of the carcinoma. This first surgery cannot really be considered as a vascularised allotransplant but more as a graft of allogenic tissue. There was no true vascular anastomosis, with the larynx only being “nursed”. It was in 1998 that Strome [6] performed the first true larynx allotransplant, but the patient’s tracheotomy tube was never removed, and the larynx had to be removed 14 years later due to chronic rejection of the graft [7]. The second published larynx allotransplant is more recent, but in this case as well the patient was still tracheotomised four years after the operation [8]. These positive results prove the feasibility of allotransplantation, but the procedure needs to be optimised to render it functional, as none of the three transplanted larynx has regained normal or subnormal mobility and the patients remained tracheotomised in the latter two cases.

The complexity of the vascularisation and innervation of this organ is responsible in part for the difficulties encountered in obtaining a reliable model for laryngeal transplantation.

Nevertheless, there is a unique and very interesting experiment that has passed completely unnoticed in the field of laryngeal transplantation, as it was only published indirectly: the article concerns the management of 13 larynx or trachea donors [9]. This Colombian team describe a reliable and reproducible model of laryngeal removal and transplantation that enables one of the major difficulties concerning the problem of vascularisation. 

The second difficulty concerns the reinnervation of the larynx, and the Colombian model does not allow an intrinsic mobility of the larynx. We have therefore conceived of a model of laryngeal transplantation combining the Colombian model and the laryngeal reinnervation model described by J.P. Marie et al. in the rehabilitation of laryngeal diplegia [10].

Thus, herein, an animal model is presented, using pigs, the animal which presents the closest anatomical similarity with the human larynx.

## 2. Materials and Methods

Six female Land Race × Large White crossed pigs, 35 kg in weight, three months of age, were used for this study with the authorisation of the Animal Welfare Committee of Nantes Veterinary School pursuant to European Directive 86/609/EEC regarding the protection of animals used for experimental and other scientific purposes.

Three laryngeal allografts were carried out with a standardised anaesthetic protocol: premedication with midazolam (0.2 mg/kg i.m.), ketamine (5 mg/kg i.m.), and medetomidine (20 μg/kg i.m.); induction with propofol (6–8 mg/kg i.v.) and analgesia with morphine (1 mg/kg i.v.); CRI fentanyl 10 μg/kg/h + ketamine 0.5 mg/kg/h and meloxicam 0.4 mg/kg.

### 2.1. Removal of the Graft

After a U-shaped (bilateral curved) cervical incision, prolonged vertically along the pre-sternal midline, the dissection started with bilateral identification of the vasculo-nervous elements: vagus and superior laryngeal nerves, the ligature of the internal jugular vein, as well as the internal carotid artery and the branches of the external carotid artery beyond the superior laryngeal artery.

**Figure 1:**
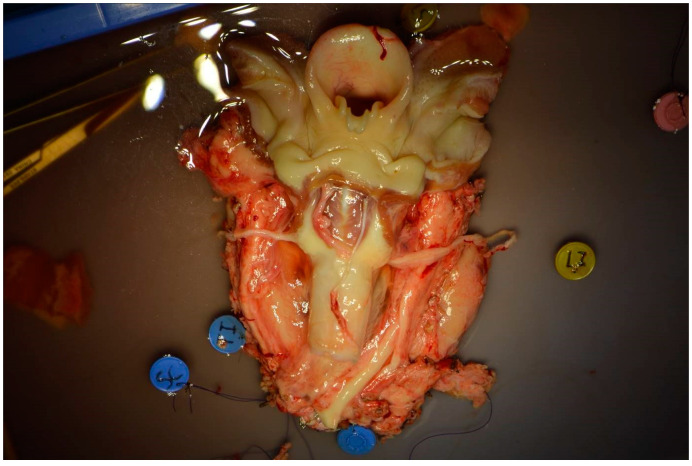
The transplant was completely harvested by performing the equivalent of a total circular pharyngolaryngectomy, including about 10 trachea rings and the entire visceral, vascular and nervous axis.

Vascular dissection continued lower down, after sternotomy, until the ascending aorta and the superior vena cava were identified. The recurrent nerves were also dissected. The ascending aorta was then canulated and the piece perfused and flushed with organ perfusion and flushing solution (Custodiol^®^, Methapharm Inc, Brantford, ON, Canada). The transplant was then completely freed by performing the equivalent of a total circular pharyngolaryngectomy, including approximately 10 trachea rings and the entire visceral, vascular, and nervous axis in the piece (Figure 1).

### 2.2. Preparation of the Graft 

The graft was then prepared in ice for transplantation. First, the vessels were chosen with opening of the brachiocephalic arterial trunk so as to identify the origin of the common carotid arteries: the trunk was retained for 1 cm below the origin of the carotid arteries so as to be able to perform an arterial anastomosis of reasonable diameter. The technique was similar for the venous axes.

The infrahyoid muscles were then resected and the hypopharyngeal mucosa prepared: section of the oesophagus, respecting the recurrent nerves, then opening of the hypopharynx respecting the mucosa of the piriform sinuses as far as possible, were carried out. The retrocricoid mucosa was then partially resected so as to cover the arytenoids and the nervous graft on the posterior cricoarytenoid muscles.

**Figure 2:**
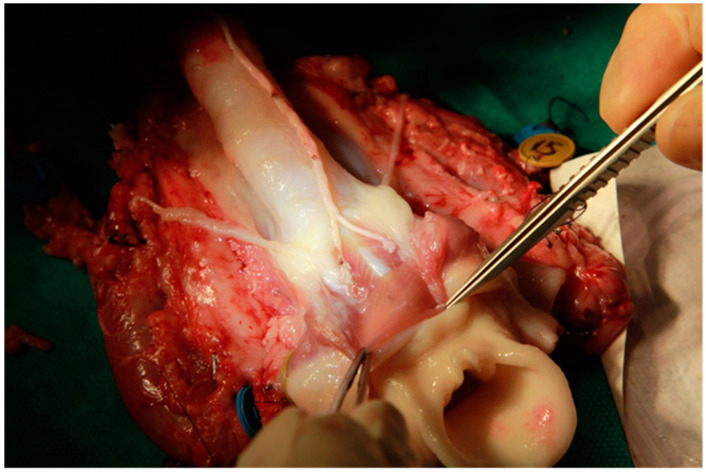
The neurological step with bilateral neurotisation of the posterior cricoarytenoid muscles using a Y-shaped graft of large auricular nerve taken from the donor.

The neurological step then began, with bilateral neurotisation of the posterior cricoarytenoid muscles using a Y-shaped graft of large auricular nerve taken from the donor (Figure 2).

### 2.3. Preparation of the Recipient

At the same time as the removal of the graft, the recipient animal was prepared for transplantation. The incision was midline at the cervical level: this was preferred to a U-shaped incision that would lead to skin necrosis in an animal. The entire arterial and venous axes were bilaterally dissected.

During the neurological step, the hypoglossal nerve and its thyrohyoid branch, the vagus nerve, and the superior laryngeal nerve were identified. The phrenic nerve was dissected higher up so as to locate the roots and anastomoses with C5.

Once all the vascular nervous elements had been identified and marked, a total laryngectomy with preservation of the hyoid bone and as much laryngeal mucosa as possible was carried out.

### 2.4. Transplantation

The posterior face of the trachea was first fixed in order to correctly fix the transplant. Then lateral sutures of the mucosae were made. The vascular phase then began by adapting the technique to the anatomy of the pig, which is variable and different to that of the human. For this study, the anastomosis performed were: for the arteries, the brachiocephalic trunk (including the two common carotid arteries) terminolaterally with the left subclavicular artery of the recipient, and for the veins, on the right and on the left, the subclavicular vein terminoterminally with the external jugular vein of the recipient.

**Figure 3:**
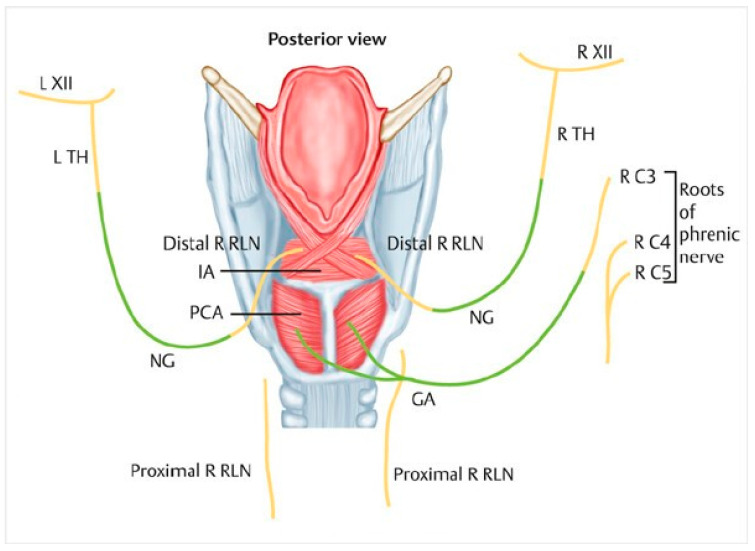
Reinnervation technique: R XII: right XII nerve; R TH: right thyro hyoid branch; NG: nerve graft; R RLN: right recurrent laryngeal nerve; PCA: posterior crico arytenoid muscle; IA: interarytenoid muscle.

After reperfusion of the graft, the reinnervation step began. The superior laryngeal nerves of the donor and of the recipient were anastomosed, as was the Y-shaped nerve transplant with the left phrenic nerve of the recipient. Finally, the recurrent nerves of the donor were anastomosed with the thyroid branch of the hypoglossal nerve on the right and on the left (Figure 3).

The suture of the hypopharyngeal mucosa, the trachea, and an inferior tracheotomy were performed at the end.

### 2.5. Post-Operative Progress

The donor animals were euthanised once the removal had been carried out (pentobarbital sodium 364.4 g i.v.).

The first two recipient animals were monitored for a few hours to verify the revascularisation, and then euthanised.

The third recipient pig was kept in monitoring for four days, awake and with a tracheotomy tube. Pharyngolaryngeal endoscopy was performed immediately after the operation and on the fourth post-operative day.

An immunosuppressant protocol (tacrolimus 0.3 mg/kg/d and prednisolone 2 mg/kg/d) and prophylactic antibiotic treatment with amoxicillin/clavulanic acid and metronidazole were administered intravenously.

## 3. Results

The mean time of intervention, including removal, preparation of the recipient and of the graft and transplantation, was 10 h 30 min (minimum 9 h 30 min, maximum 11 h 40 min).

The cold ischaemia time (time between flushing and the start of anastomoses) of the graft was 2 h on average, and the warm ischaemia time (time between the start of vascular anastomoses and the clamping) was 1 h 5 min. Results are shown in Table 1.

The tolerance of anaesthesia was excellent for the six pigs, both donors and recipients.

The different phases of the transplantation could be respected in all cases, and adaptation to the inter-individual anatomical variability did not constitute an obstacle. The transplantation protocol was correctly and reproducibly followed in the three operations.

**Figure 4:**
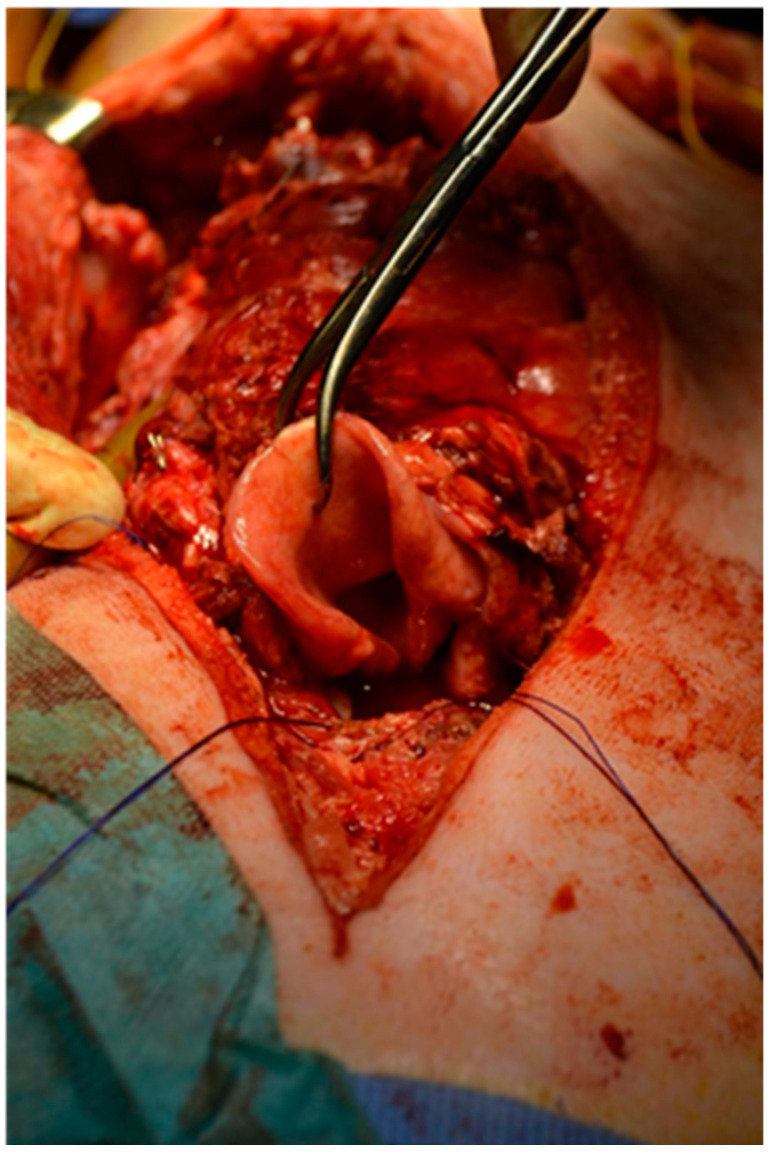
Once the anastomoses had been carried out, the vessels were de-clamped and perfect in vivo revascularisation of the transplant could be observed after a few minutes.

Once the anastomoses had been carried out, the vessels were de-clamped and perfect in vivo revascularisation of the transplant could be observed after a few minutes (Figure 4).

**Figure 5:**
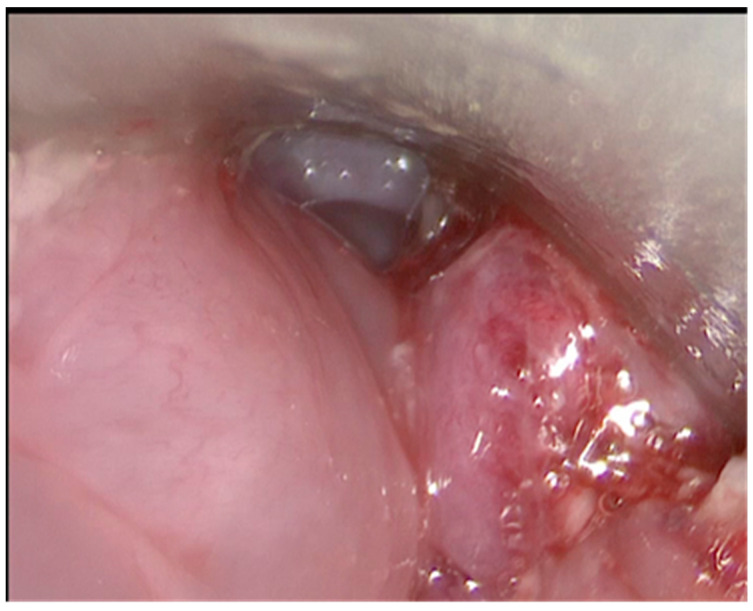
Endoscopy immediately performed after the surgery in the third recipient animal confirmed the correct revascularisation of the transplant.

An endoscopy was immediately performed after the surgery in the third recipient animal and confirmed the correct revascularisation of the transplant (Figure 5).

The animal was monitored for four days with no significant problems. Endoscopy on the fourth day confirmed the viability of the transplant. The animal died on the fifth day after the operation, and the autopsy revealed massive mesenteric infarction.

## 4. Discussion

The complexity of the laryngeal vascularisation and innervation is probably one of the reasons explaining the small number of larynx transplants published to date. Furthermore, to our knowledge, there is no real description of larynx removal that would allow the results that have been published to date to be reproduced [4,6]. It seemed important to us to obtain a reproducible model of larynx transplant before undertaking a transplantation program in humans.

The pig model seemed the most appropriate to us; it has also been chosen in particular by Birchall et al., who also showed its reliability [11]. In comparison with the rat [12], the anatomical elements of the pig are closely comparable to those of humans, which also allows the reinnervation technique to be studied [13,14,15]. The dog has also been chosen, especially in the earliest work on allotransplantation, but the morbi-mortality of this model was high [16,17,18] and its current use in animal experimentation is limited for ethical reasons.

Furthermore, even if our objective was to evaluate the feasibility and reproducibility of the technique that we have developed, the good tolerance to immunosuppression in pigs allows immunological studies, as shown by studies for the transplantation of other organs, in particular bronchi and trachea [19,20].

In our experience, the choice of the pig as an animal model enables the transplantation model to be as close as possible to humans, thanks to the close anatomical similarities. The variations, particularly in the vascular network, did not hinder the revascularisation of the transplant, in part due to the single-block removal up to the ascending aorta and the vena cava. This animal model shows the feasibility and reproducibility, in terms of vascularisation, of this laryngeal removal and transplantation technique. We were able to obtain three transplanted vascularised larynges, to at one hour after the operation and the third at four days after the operation. Preferring large vessels for the vascular anastomoses enables a perfect viability of the transplant to be ensured.

The principal objective of this work was not concerned with the reinnervation because the first results, either in terms of reinnervation or respiration to be able to envisage removal of the tracheotomy tube, would only be visible after several months. This period seemed to us to be technically, ethically, and financially incompatible with keeping a transplanted and tracheotomised animal alive. Nevertheless, the anatomical similarities between the pig model and humans enabled us to carry out the nervous anastomoses, which is important in the progress of the different steps of a transplantation. This allowed us to model a complete transplantation in both vascular and neurological terms and so to precisely determine the order of the different operative steps.

This original technique of laryngeal reinnervation was inspired by the work of J.P. Marie et al. on laryngeal diplegia [21,22]. Marie et al. propose a neurotisation of the posterior cricoarytenoid muscles by a Y-shaped graft of the large auricular nerve. This graft is then unilaterally micro-anastomosed with the superior root of the phrenic nerve without significantly affecting diaphragm function and without respiratory impact. In this way, the dilator muscles of the glottis are stimulated on inhalation. At the same time the adductor muscles are re-innervated by the thyrohyoid branch of the hypoglossal nerve, for contraction on swallowing, in a bilateral fashion so as to avoid erroneous reinnervation of these adductor muscles by branches of the phrenic nerve. The identification of the thyrohyoid branch is difficult and requires a certain amount of practice.

This technique, which has shown its utility in the treatment of laryngeal diplegia [23], seems applicable to laryngeal transplantation in our model. It constitutes one of the innovative elements, compared to techniques previously described in the literature, aiming to obtain a functional larynx. This allows us to envisage the possibility of larynx transplant with a mobile larynx and patients without a tracheotomy tube, something which, to our knowledge, has never been carried out to date. The studies of reinnervation for bilateral recurrent paralysis have shown that the selective reinnervation via the phrenic nerve allows sufficient laryngeal opening to envisage the removal of the tracheotomy tube and even preservation of the quality of the voice [8]. This technique is therefore an alternative to the arytenoidopexy performed by the Colombian team (unpublished results).

## 5. Conclusions

With the technique of removal in a single block and anastomoses with large vessels, we can consider that this animal model of laryngeal transplantation is reliable and reproducible with regard to revascularisation. These results encourage us to continue this work in order to consider offering this technique to humans in the near future.

## Figures and Tables

**Table 1:** Operative time, ischemia times, tolerance and post-operative revascularisation assessment for the three transplantations.

	Transplantation 1	Transplantation 2	Transplantation 3	Mean Time
Operative time	570 min	620 min	700 min	630 min
Cold ischemia	140 min	115 min	105 min	120 min
Warm ischemia	60	65	70	65 min
Anesthesia tolerance	Excellent	Excellent	Excellent	
Post operative revascularisation assesment	none	none	Endoscopic at day 0 and day 4	
